# Representational horizon and visual space orientation: An investigation into the role of visual contextual cues on spatial mislocalisations

**DOI:** 10.3758/s13414-023-02783-5

**Published:** 2023-09-20

**Authors:** Nuno Alexandre De Sá Teixeira, Rodrigo Ribeiro Freitas, Samuel Silva, Tiago Taliscas, Pedro Mateus, Afonso Gomes, João Lima

**Affiliations:** 1 William James Center for Research, University of Aveiro, Campus Universitário de Santiago, 3810-193 Aveiro, Portugal; 2 Department of Education and Psychology, University of Aveiro, Aveiro, Portugal; 3 Institute of Electronics and Telematics Engineering of Aveiro (IEETA), Intelligent Systems Associate Laboratory (LASI), Department of Electronics, Telecommunications and Informatics (DETI), University of Aveiro, Aveiro, Portugal

**Keywords:** Representational Momentum, Representational Gravity, Representational Horizon, Motion perception, Spatial orientation

## Abstract

The perceived offset position of a moving target has been found to be displaced forward, in the direction of motion (*Representational Momentum*; RM), downward, in the direction of gravity (*Representational Gravity*; RG), and, recently, further displaced along the horizon implied by the visual context (*Representational Horizon*; RH). The latter, while still underexplored, offers the prospect to clarify the role of visual contextual cues in spatial orientation and in the perception of dynamic events. As such, the present work sets forth to ascertain the robustness of *Representational Horizon* across varying types of visual contexts, particularly between interior and exterior scenes, and to clarify to what degree it reflects a perceptual or response phenomenon. To that end, participants were shown targets, moving along one out of several possible trajectories, overlaid on a randomly chosen background depicting either an interior or exterior scene rotated −22.5º, 0º, or 22.5º in relation to the actual vertical. Upon the vanishing of the target, participants were required to indicate its last seen location with a computer mouse. For half the participants, the background vanished with the target while for the remaining it was kept visible until a response was provided. Spatial localisations were subjected to a discrete Fourier decomposition procedure to obtain independent estimates of RM, RG, and RH. Outcomes showed that RH’s direction was biased towards the horizon implied by the visual context, but solely for exterior scenes, and irrespective of its presence or absence during the spatial localisation response, supporting its perceptual/representational nature.

The transmission of neural impulses, and, hence, information relay, is known to be relatively sluggish, a fact first noticed by von Helmholtz in 1850 (Warren & Warren, 1968). For visual afference, these delays might add up to about 100 milliseconds, between the cells in the retina until the striate cortex, which would impair our abilities to accurately perceive a moving object, let alone prepare and execute appropriate motor actions (see Nijhawan, 2008). The very fact that, *ceteris paribus*, simply intersecting a moving ball is uneventful in our daily lives and, generally, taken for granted within our behavioural repertoire, suggests that the perception of a dynamic event comprises extrapolation-neural mechanisms which actively compute its future states (Hogendoorn, 2020; Nijhawan, 1994, 2002, 2008). On the other hand, it has been suggested that such extrapolation mechanisms are further fine-tuned by explicitly considering ecologically relevant environmental invariants (Shepard, 1984), such as, prominently, the constant gravitational acceleration on Earth, in the form of an analogous internal model (Angelaki et al., 2004; Grush, 2005; Lacquaniti et al., 2013; McIntyre et al., 2001; Tin & Poon, 2005). Finally, and given that humans, as well as other animals, are themselves moving beings, the perceived direction of gravity has to be continuously updated and is known to depend on an integration of vestibular, somatosensory (idiotropic vector), and visual cues (Barra et al., 2010; Haji-Khamneh & Harris, 2010; Harris et al., 2011; Howard & Templeton, 1973; Jenkin et al., 2011; Kheradmand & Winnick, 2017; MacNeilage et al., 2008; Mittelstaedt, 1986; Oman, 2007; Volkening et al., 2014).

These lines of reasoning have come to intersect in the psychophysical research on spatial mislocalisation phenomena of moving stimuli. In their seminal study, Freyd and Finke (1984) reported that after observers were shown a sequence of images depicting a rectangle undergoing apparent rotation, they were more prone to judge that a static rectangle had the same orientation as the last one in the inducing sequence if it was, actually, further rotated in the direction of implied motion. In the original interpretation, and upon seeing a kinematic stimulus, a contingent visual representation is automatically generated, endowed with dynamic features which analogously represent temporal and physical invariants—*Dynamic Representations* (Freyd, 1987). When the inducing stimulus is halted, the accompanying dynamic representation keeps unfolding for some time, resulting in a misjudgement of the actual last seen orientation, as if an analogue of momentum was embedded in the visual representation—*Representational Momentum* (for an early review, see Hubbard, 2005). In accordance, bigger Representational Momentum was found for faster shown motions (Finke et al., 1986; Freyd & Finke, 1985) and for increasing temporal intervals imposed after motion offset, until a maximum at about 300 ms (Freyd & Johnson, 1987). Further research revealed Representational Momentum to be affected by tacit knowledge regarding expected dynamics—for instance, it was found to be increased for kinematic displays depicting an ascending rocket, in comparison with a building (with similar visual features; Reed & Vinson, 1996), and to emerge for static freeze-frame photographs implying motion (e.g., a person jumping from a wall; Freyd, 1983). Similarly, and of relevance, Representational Momentum was also found to be increased for objects moving downward, in the direction of gravity (Bertamini, 1993; Nagai et al., 2002).

Ever since, Representational Momentum has been replicated with varying types of stimuli and different paradigms (Hubbard, 2005, 2015), including, prominently, continuous moving targets and motor spatial localisation responses (Hubbard, 1990, 1995a, b; Hubbard & Bharucha, 1988). In these studies, a simple target (e.g., a square or a circle) is shown moving smoothly along the screen and, after covering a certain distance, disappears. Afterwards, a cursor controllable with a computer mouse or a trackball appears on the centre of the screen, and participants are instructed to position it at the location where the target vanished. With this method, and due to the bidimensionality of the spatial localisation onscreen, it is possible to compute not only the displacement along the target’s trajectory—*M-displacement*—but also the displacement orthogonal to the target’s trajectory—*O-displacement* (Hubbard, 1998). For horizontally moving targets, and besides a forward M-displacement, indexing the classical Representational Momentum, a downward O-displacement is also systematically found. Furthermore, for vertically moving targets, O-displacement is usually null, but the forward M-displacement is significantly bigger for descending targets than for ascending ones. Both the downwards O-displacement for horizontally moving targets and the directional asymmetry found for M-displacement with vertically moving targets have been interpreted as an empirical measure of *Representational Gravity* (Hubbard, 1990, 1995a, 1998, 2020; Hubbard & Bharucha, 1988), a putative mental analogue of gravity. Theoretically, these types of spatial mislocalisation phenomena have been taken as an empirical manifestation of extrapolation perceptual mechanisms (Hubbard, 2005, 2015; Vinson et al., 2017) which reflect internalized ecologically relevant physical invariants, extending the notion of second-order isomorphism paved out by Roger Newland Shepard (1984, 1994), and, recently and particularly in what refers to Representational Gravity, an internal model of gravity (Angelaki et al., 2004; Barra et al., 2010; De Sá Teixeira, 2014; Grush, 2005; Lacquaniti et al., 2013; McIntyre et al., 2001; Tin & Poon, 2005).

Notwithstanding, alternate accounts for Representational Momentum, particularly for smoothly moving targets, reliant upon low-level mechanisms, have been put forth, prominently, by Kerzel (2000, 2006). Briefly, when shown a smoothly moving object, observers typically track it with their eyes, engaging smooth pursuit eye movements which tend to overshoot its offset when it suddenly halts (Mitrani & Dimitrov, 1978; Pola & Wyatt, 1997). This oculomotor feature might arguably explain the forward displacement found in Representational Momentum studies, precluding the need to postulate a role for high-level cognitive-based mechanisms (Kerzel, 2006). In agreement, Representational Momentum for continuously moving targets (but not implied motion stimuli; Kerzel, 2003) has been found to be null, or severely reduced, when smooth pursuit eye movements are prevented (e.g., by requiring observers to fixate a point; De Sá Teixeira et al., 2013; De Sá Teixeira, 2016b; De Sá Teixeira & Oliveira, 2014; Kerzel, 2000; Kerzel et al., 2001), although that reduction does not seem to be the case when participants have to manually point to the perceived offset location (Ashida, 2004; Kerzel & Gegenfurtner, 2003). Concurrently, preventing smooth pursuit eye movements seems to have no discernible effect on Representational Gravity—neither the increased forward M-displacement for descending targets (De Sá Teixeira, 2016b) nor the downward O-displacement for horizontally moving targets (De Sá Teixeira et al., 2013; De Sá Teixeira & Oliveira, 2014) are affected by preventing eye movements or, for that matter, by response modality (De Sá Teixeira et al., 2019a, b). Furthermore, and regardless of the presence or absence of eye movements, Representational Gravity continuously increases as longer temporal intervals are imposed between target offset and spatial localisation response initiation (De Sá Teixeira, 2016a, b; De Sá Teixeira et al., 2013; De Sá Teixeira & Hecht, 2014), in a pattern that further differentiates it from Representational Momentum.

Notice that, depending on the direction of the target’s motion, Representational Momentum and Representational Gravity might be more or less entangled, jointly determining the spatial mislocalisation (for a detailed geometrical derivation of the periodic changes in M-displacement as the orientation of target’s trajectory is varied see De Sá Teixeira, 2016b; see also Hubbard, 1995a, 2005, 2020). Specifically, consider a descending target—in this case, both Representational Momentum and Representational Gravity would lead to an increased M-displacement along the same vector (downwards); for an ascending target, the reverse is the case, with Representational Momentum and Representational Gravity acting in opposite directions and partially cancelling each other out in the measurement of M-displacement; finally, for horizontally moving targets, Representational Momentum alone would determine the magnitude of M-displacement (with Representational Gravity reflecting solely on O-displacement). This logic can be further expanded—consider a target moving diagonally: in this case, M-displacement, in addition to the contribution of Representational Momentum, should be slightly increased or decreased (depending on whether the vertical component of the target’s motion is, respectively, downward or upward) as it would be partially affected by Representational Gravity. Stated differently, Representational Momentum is made manifest by a forward M-displacement, irrespective of target’s motion direction, the magnitude of which is further modulated by Representational Gravity, made manifest by a further displacement along one single direction (downwards). Analytically, the specific contribution of Representational Gravity (and, by extension, its magnitude and direction) can be neatly determined as a periodic component embedded in a set of M-displacements, if and when measured for targets moving along several directions within the frontal-parallel plane, by taking advantage of the Fourier theorem (with target’s motion direction as parameter; for an in depth explanation of the underlying logic and procedure of the Fourier decomposition see Sekuler & Armstrong, 1978).

Using this logic, it was found that the direction of Representational Gravity coincides with whichever direction in which the participants’ feet are pointed, although its time course is reduced as participants’ bodies are further misaligned with the gravito-inertial vector (that is, Representational Gravity becomes a constant and does not increase with time; De Sá Teixeira, 2014; De Sá Teixeira et al., 2017), reflecting the contribution of vestibular processing (De Sá Teixeira et al., 2019a, b). Furthermore, besides Representational Momentum and Representational Gravity, these studies systematically reported one further periodic component, accounting for an increased M-displacement for targets moving horizontally (either rightwards and leftwards). The relevance of this latter harmonic component has only recently been ascertained, though. Freitas and De Sá Teixeira (2021) conducted a study where M-displacement was measured for targets moving along 16 possible directions (leftward, rightward, downward, upward, and varying degrees of diagonal trajectories in between) while overlaid on a background image depicting the interior view of the Harmony module of the International Space Station, which could be either on an upright orientation or tilted leftwards or rightwards by 22.5º. The second harmonic’s orientation was found to be biased towards the horizon implied by the visual context—that is, targets moving along the horizontal line implied by the background scene (irrespective of its misalignment with the actual horizontal) led to increased M-displacements. This trend was further found to be correlated, at an individual level, with measures of subjective visual vertical (SVV) made with the same visual context, strengthening the relevance of visual spatial orientation (Haji-Khamneh & Harris, 2010; Harris et al., 2011; Howard & Templeton, 1973; Jenkin et al., 2010, 2011; MacNeilage et al., 2008; Oman, 2007) for the perception of dynamic events (see also Moscatelli & Lacquaniti, 2011).

In this vein, the present study has as its primary objective to replicate the results of Freitas and De Sá Teixeira (2021), specifically in what refers to the effect of visual context orientation on the second harmonic present in M-displacements—which we coin *Representational Horizon*—and to extend that finding for a variety of scenes, beyond the one used in that previous study. As a secondary goal, we set forth to ascertain to what degree Representational Horizon is determined by the presence of the visual context during the spatial localisation response or if it only requires that the inducing moving stimuli is shown embedded in it. The former scenario would cast doubts that Representational Horizon reflects the perceptual processing of the dynamic event, while the latter would support that view. Finally, we sought to test the robustness of the effect of the orientation of the visual context on Representational Horizon by using both interior scenes (e.g., bedrooms, living rooms, halls, libraries) and exterior scenes (e.g., rural and urban landscapes, beaches, streets, forests). To that end, we performed a standard spatial localisation task for the offset position of a target that could be shown moving along several directions, overlaid on an upright or tilted (leftward or rightward) visual context, depicting either an interior or exterior scene. For half of the participants, the visual context shown while the target moved remained onscreen until a spatial localisation response was provided; for the remainder of the participants, the visual context was replaced with a blank screen (with the same mean luminance as the visual context) when the target vanished.

## Method

### Participants

Based on the reported magnitude of the effect of visual context orientation (partial η^2^ = 0.21, for the coefficient *b*_*2*_) in Freitas and De Sá Teixeira (2021), a power analysis reveals that a minimum sample size of 12 participants would be required. To strengthen the robustness of statistical inference, forty participants (29 females; 11 males) were recruited for the experiment in exchange for partial course credits. Their ages ranged from 18 to 26 years (*M* = 19.8 years, *SD* = 1.58) and all had normal or corrected-to-normal vision and no known neurological or vestibular deficits. The experiment was preapproved by the Ethics Committee of the University of Aveiro (Protocol 34-CED/2021).

### Stimuli

**Fig. 1:**
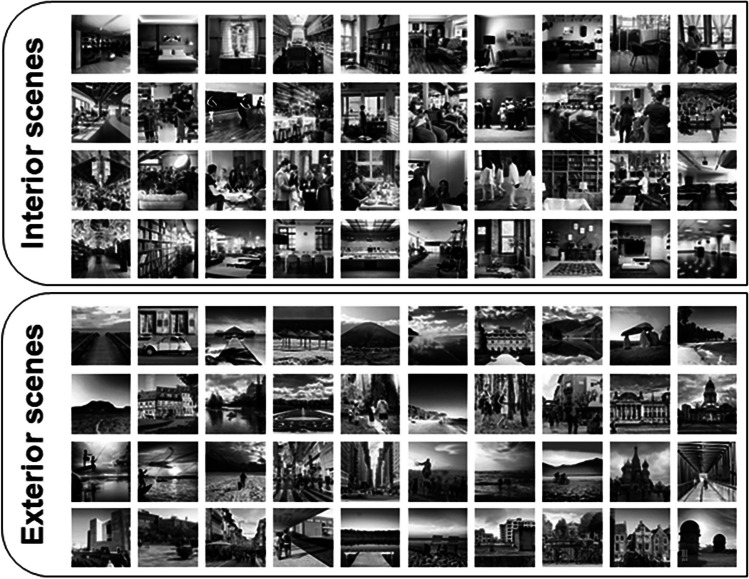
Image pool used for visual context, separated by interior (top panel) and exterior (bottom panel) scenes

Eighty free stock images were used as visual context (see Fig. 1), 40 depicting exterior scenes (e.g., beaches, mountains, urban and rural landscapes) and 40 depicting interior scenes (e.g., bedrooms, living rooms, libraries, gymnasiums, halls). These images were chosen to be as varied as possible and rich in visual orientation cues. The selected images were processed as follows: each image was cropped to conform to a 1:1 ratio and its size adjusted to the height of the screen (1,024 pixels); afterward, all images were rendered as black-and-white and their luminance equalised to RGB = (127, 127, 127). The target for the spatial localisation task was a black circle, with a radius of 21 pixels (about 0.7º of visual angle) with a white circumference of 1 pixel.

### Apparatus, procedure, and design

Participants sat in an office chair, with adjustable height, in front of a computer screen with a refresh rate of 60 Hz and resolution of 1,280 × 1,024 pixels (physical size of 37.5 × 30 cm). Their view of the screen was restricted to a circular central window with a custom-made black cardboard cylinder with a diameter of 30 cm (equal to the height of the screen) and a length of 50 cm (ensuring a fixed distance between the participant’s cyclopean eye and the centre of the screen in addition to occluding any peripheral view of the laboratory setting). Furthermore, participants wore noise-cancelling earmuffs during the experimental task, to minimise distracting noises from the laboratory and/or building. Stimuli presentation, trial randomization, and data collection were programmed in Python using PsychoPy (Peirce, 2007, 2008).

**Fig. 2:**
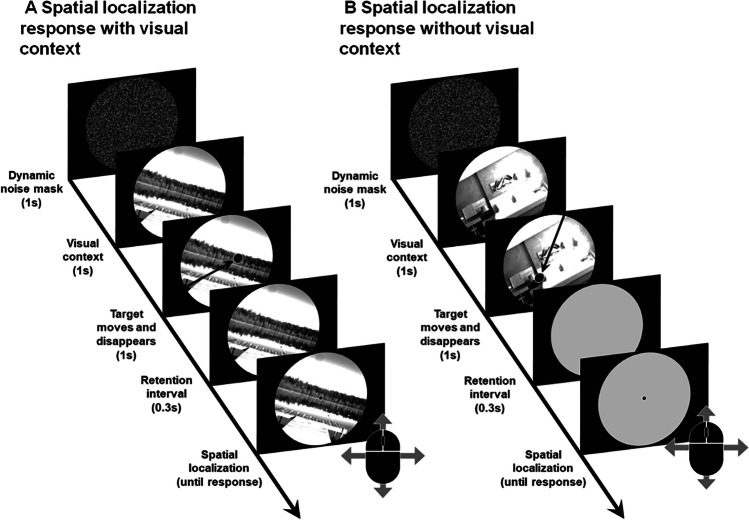
Trial structures when spatial localisation responses are made with (**A**) and without (**B**) the same visual context present during the target’s motion

Each trial (see Fig. 2) started with a random sequence of noise frames during 1 second, being immediately replaced with a randomly chosen image (visual context) from one of two image pools (interior or exterior scenes), covering the entire visible section of the screen and either tilted leftward (−22.5º), rightward (22.5º), or in an upright orientation (0º). One second after the onset of the visual context, the target emerged from the visible boundary of the circular window, already in motion toward the centre of the screen at a speed of 616 pixels/s (about 20.4º/s). The target’s motion lasted for 1 second, and it could be shown moving leftward (trajectory orientation of 0º), rightward (180º), upward (90º), downward (270º), or intermediate trajectories between those cardinal orientations (22.5º, 45º, 67.5º, 112.5º, 135º, 157.5º, 202.5º, 225º, 247.5º, 292.5º, 315º, or 337.5º). The target’s starting location was randomly chosen such that its offset fell inside an area of 16 × 16 pixels (about 0.54º × 0.54º) centred 92 pixels beyond the screen’s centre (about 3.1º). Upon the vanishing of the target, the visual context either remained on the screen (for half the participants) or was immediately replaced by a blank grey screen (RGB = [127, 127, 127]; for the remainder of the participants) until a response was given. In both cases, 300 ms later a cursor, given by a black dot with a diameter of 5 pixels with a 1-pixel white contour, appeared on the centre of the screen. The cursor’s location onscreen was controllable with a computer mouse and the participants were instructed, at the beginning of the experiment, to use it to position the cursor in the same location where the target vanished, as precisely as possible and referring to its geometric centre. The spatial location was confirmed by pressing the left mouse button. The next trial started 500 ms after each spatial localisation response.

The experiment thus followed a mixed factorial design given by 3 (visual context orientation: −22.5º, 0º, or 22.5º) × 2 (visual scene: interior or exterior) × 16 (orientation of target trajectory: 0º, 22.5º, 45º, 67.5º, 90º, 112.5º, 135º, 157.5º, 180º, 202.5º, 225º, 247.5º, 270º, 292.5º, 315º, or 337.5º) × 4 (replications) × 2 (response background: blank screen or visual context; between-participants), totalling 384 trials per participant. Before the experiment, the participants performed a few practise trials until the experimenter made sure the task was fully understood. Finally, a brief pause was allowed after half of the experimental trials were completed. The entire experiment, including instructions, debriefing, practise trials, main task, and intermission, lasted about 80 minutes.

### Calculations, hypotheses, and statistical analyses

For each trial, the horizontal and vertical difference, in pixels, between the target’s actual offset and the location indicated by the participant was calculated. The obtained values were then used to calculate the orthogonal projection of the participant’s response onto the target’s motion trajectory, to obtain the displacement along motion direction—M-displacement—and such that positive values indicate a displacement forward and negative values a displacement backwards, in relation to motion direction. The individual sets of M-displacements, averaged across replications and for each experimental condition, were subjected to a discrete Fourier decomposition (for a detailed tutorial on this procedure, see Sekuler & Armstrong, 1978; see also De Sá Teixeira, 2014, 2016b; Freitas & De Sá Teixeira, 2021), with target’s motion direction (*θ*) as parameter, and so as to obtain the individual estimates of a constant *c* and harmonic coefficients *a*_*i*_ (cosine) and *b*_*i*_ (sine) up to *i* = 4, in accordance with:

1 $${M}_{\theta }=c+{\sum\nolimits }_{i=i}^{n}\left({a}_{i}\mathrm{cos}\;i\frac{\theta }{2\pi }+{b}_{i}\mathrm{sin}i\frac{\theta }{2\pi }\right).$$

**Fig. 3:**
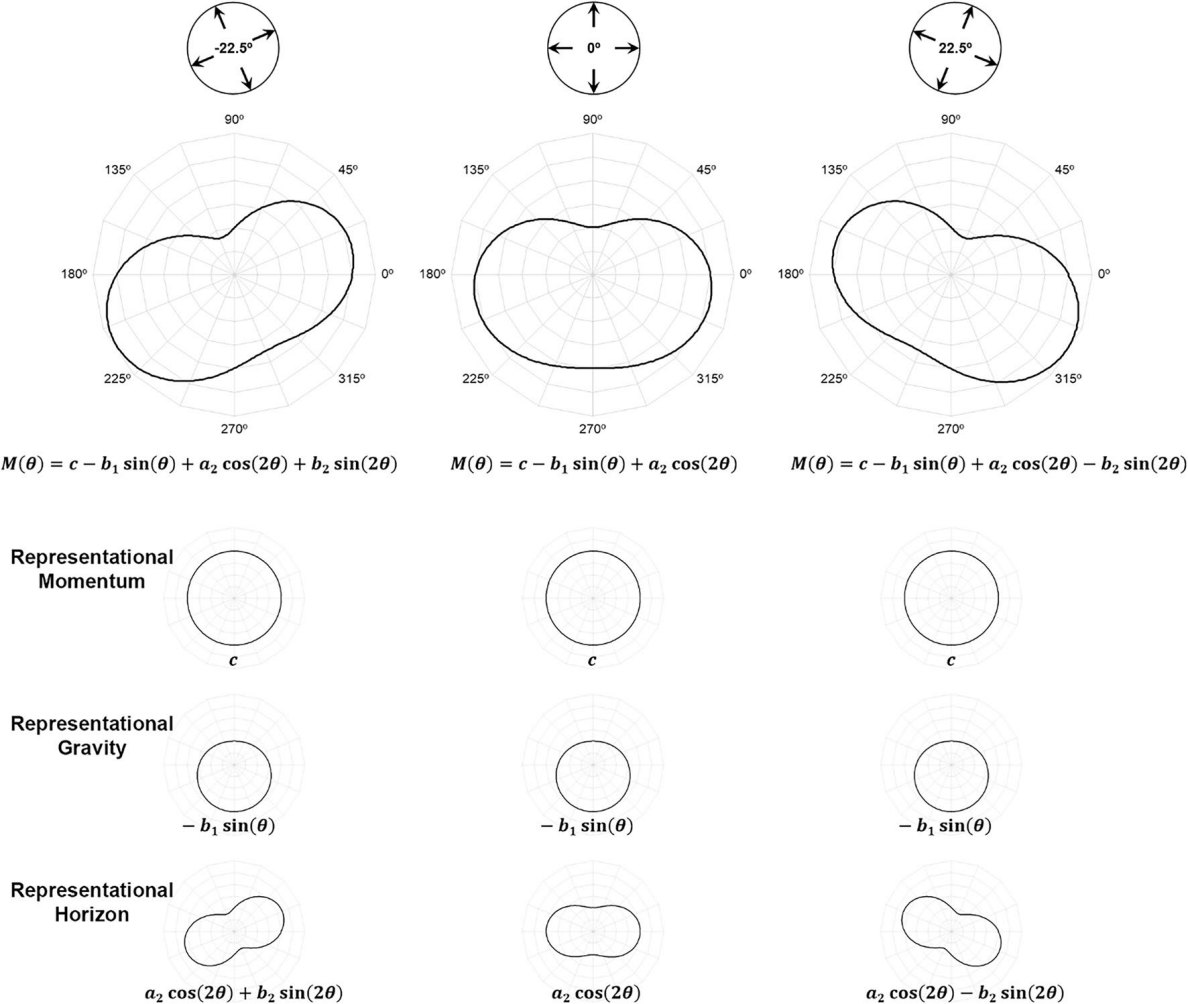
Hypothesized effect of visual context orientation on M-displacements, depicted in polar plots. *Note.* The bottom insets represent the underlying harmonic components for each visual context orientation

In Eq. 1, *c* reflects a constant displacement across all motion directions *θ* and, as such, is taken as a measure of Representational Momentum (see Fig. 3, first inset line). Coefficients *a*_*1*_ and *b*_*1*_, taken together, reflect an increased displacement towards one preferred direction; in previous studies (De Sá Teixeira, 2016b; Freitas & De Sá Teixeira, 2021), *a*_*1*_ was found to be null and a negative *b*_*1*_ is commonly found, which translates as a greater displacement downward, towards the participants’ feet and, thus, taken as a measure of Representational Gravity (see Fig. 3, second inset line). The coefficient *a*_*2*_ is typically found to be significant, reflecting an increase in forward displacement for horizontally moving targets—Representational Horizon. Importantly, it has been previously reported (Freitas & De Sá Teixeira, 2021) that the orientation of the visual context is accompanied by a modulation of coefficient *b*_*2*_ such that the orientation of Representational Horizon is biased toward the horizon implied by the visual context (see Fig. 3, third inset line). In that same study, coefficients *a*_*4*_ and *b*_*4*_—which together reflect an increased forward displacement along four preferred directions—were also found to be affected by the orientation of the visual context, although this trend is arguably due to the fact that the specific visual context employed depicts a prominent rectangular frame. Given the set of images used as visual context in the present study, we hypothesized that varying orientations of the visual context would reflect solely on the measured *b*_*2*_ coefficients (see Fig. 3, top plots).

To statistically test these hypotheses, estimated individual values of *c*, coefficients *a*_*1*_–*a*_*4*_ and *b*_*1*_–*b*_*4*_, were subjected to a mixed multivariate analysis of variance (MANOVA), with response context (inducing stimuli or blank screen) as a between-subjects factor and visual context orientation (−22.5º, 0º, and 22.5º) and scene type (interior or exterior scenes) as repeated-measures factors. Whenever the sphericity assumption was violated, degrees of freedom were adjusted with the Greenhouse–Geisser correction.

## Results

**Fig. 4:**
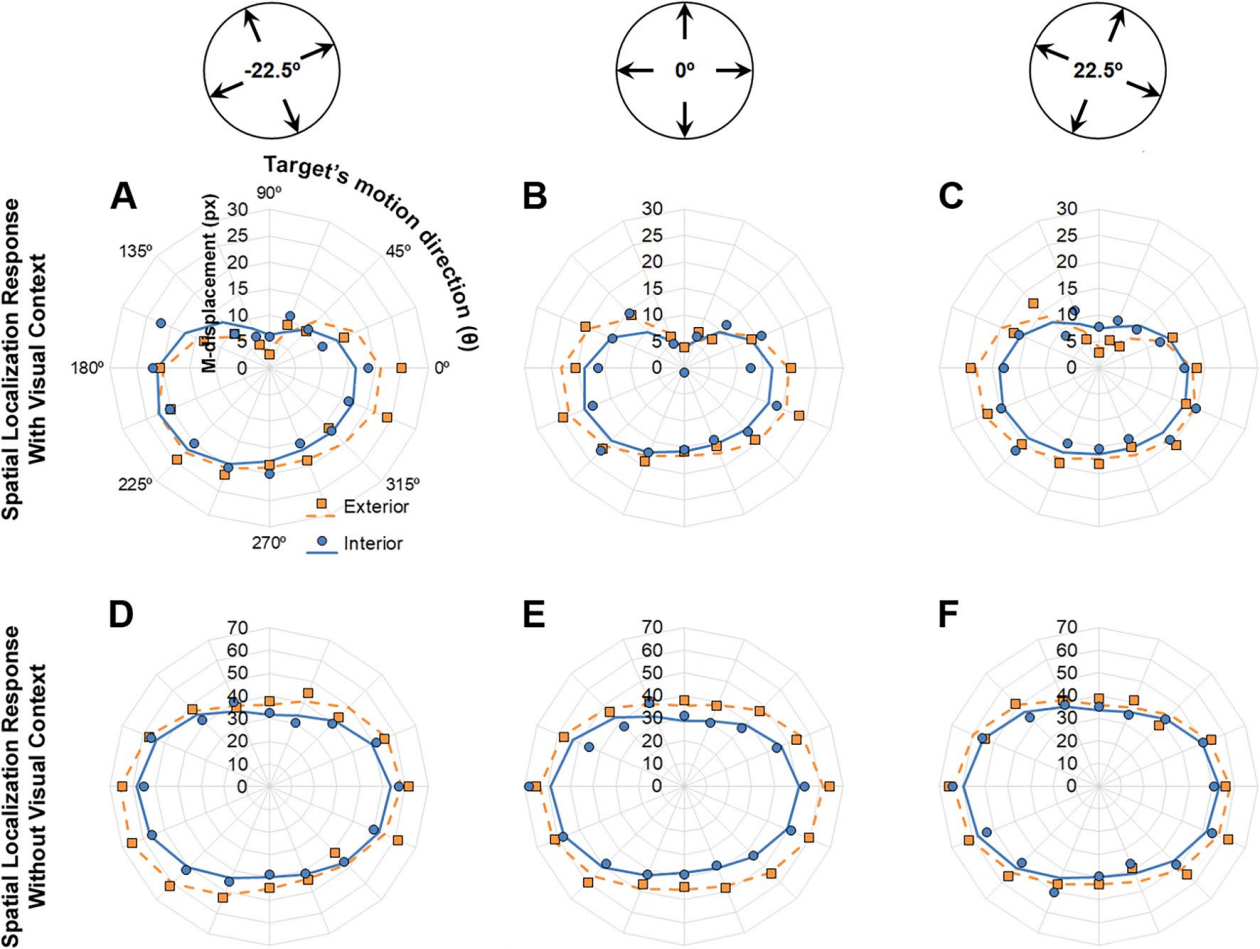
Polar plots of M-displacements as a function of target’s motion direction (θ; radial parameter). *Note.* Data markers depict empirically observed *M-displacements* and lines the best fitting models including constant *c* (RM), coefficients *a*_*1*_ and *b*_*1*_ (RG), and coefficients *a*_*2*_ and *b*_*2*_ (RH). Top insets depict the orientations of visual context

Prior to the main analyses, mean M-displacement for each target’s motion direction, visual context orientation and scene type were subjected to one-sample *t* tests to ensure that it differed from 0 and, hence, that forward perceptual displacements were observed. For all conditions, M-displacement was significantly bigger than zero (*p* < .002 for all tests). Figure 4 depicts polar plots for the mean M-displacements as a function of target’s motion direction (radial lines) and scene type (line parameter) for the varying orientations of the visual context (panel columns) and presence/absence of a visual context during the spatial localisation responses (line parameter). Visual inspection shows that M-displacements were considerably higher when no visual context was shown during the response stage (plots D, E, and F). Also, M-displacements seem to vary with the orientation of the visual context, being somewhat larger for targets moving along the horizon implied by the visual context.

**Fig. 5:**
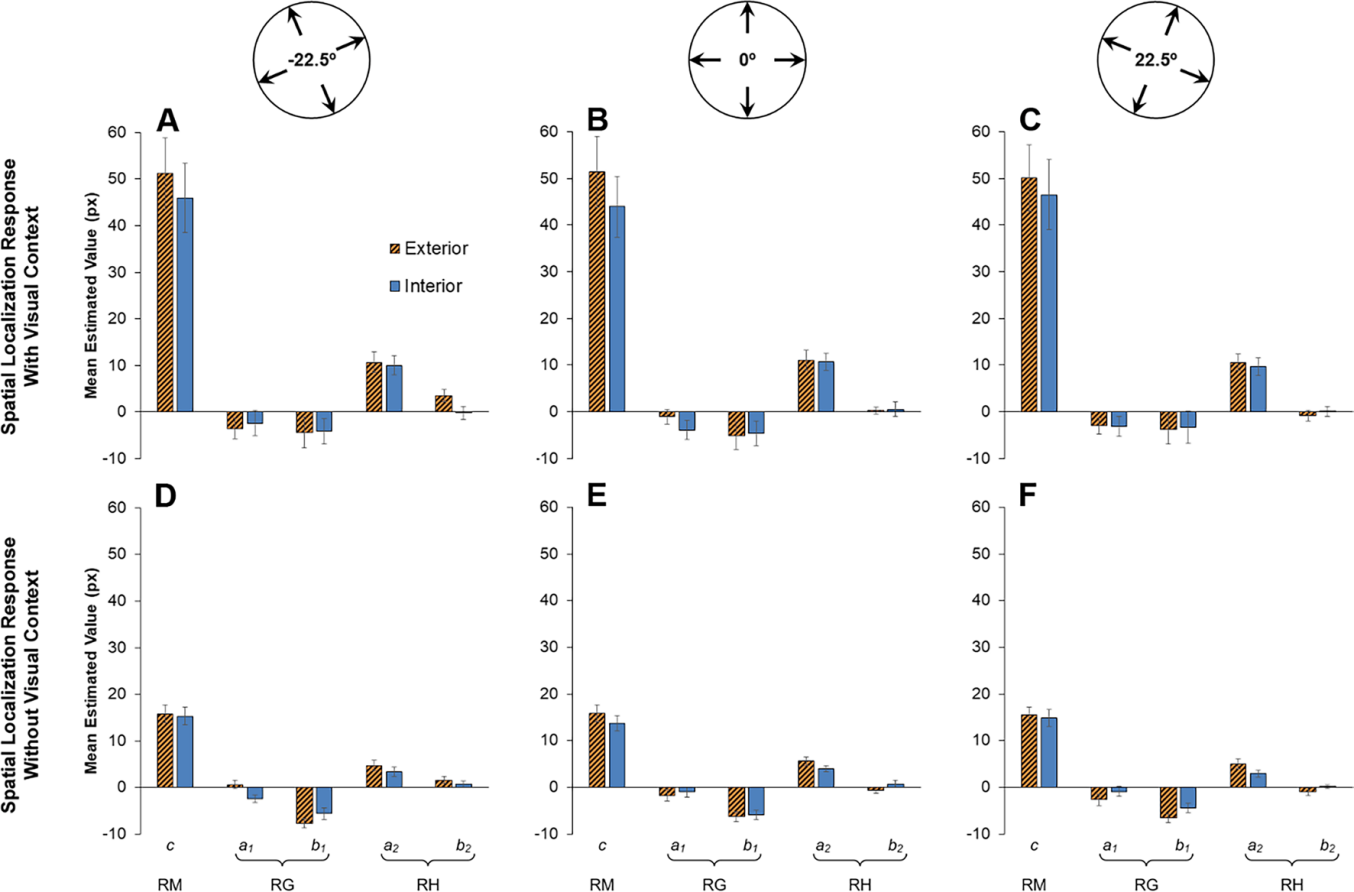
Mean estimated constant *c* (Representational Momentum; RM), coefficients *a*_1_ and *b*_1_ (Representational Gravity; RG), and coefficients *a*_2_ and *b*_2_ (Representational Horizon; RH). *Note.* Error bars depict the standard errors of the means. Top insets depict the orientations of visual context

Statistical analyses provided support for visual inspection. Response context significantly affected constant *c*, *F*(1, 38) = 19.67, *p* < .001, partial η^2^ = 0.341, coefficient *a*_*2*_, *F*(1, 38) = 10.75, *p* = .002, partial η^2^ = 0.221, and coefficient *b*_*3*_, *F*(1, 39) = 4.393, *p* = .043, partial η^2^ = 0.104. Overall, spatial localisation responses made with a blank background led to a considerably higher Representational Momentum (constant *c*: *M* = 48.21, *SD* = 32.33), compared with responses made with a visual context (constant *c*: *M* = 15.17, *SD* = 7.99; see Fig. 5). At the same time, responses made with a blank background led to both a slightly higher *a*_*2*_ coefficient (*M* = 10.45, *SD* = 7.92) and a negative *b*_*3*_ coefficient (*M* = −1.31, *SD* = 2.32), compared with responses made with a visual context (*a*_*2*_: *M* = 4.24, *SD* = 2.97; *b*_*3*_: *M* = −0.15, *SD* = 0.86), which, taken together, reflect a larger forward displacement in the former condition for targets moving along the actual horizontal and in a downwards slant. No other main effects were found for the response context.

In what refers to the effects of visual context, scene type was found to significantly affect constant *c*, *F*(1, 38) = 48.038, *p* < .001, partial η^2^ = 0.558, disclosing a bigger Representational Momentum for exterior scenes, and more so for those participants whose responses were made without visual context, as revealed with a significant interaction between scene type and response context, *F*(1, 38) = 21.401, *p* < .001, partial η^2^ = 0.36.

**Fig 6:**
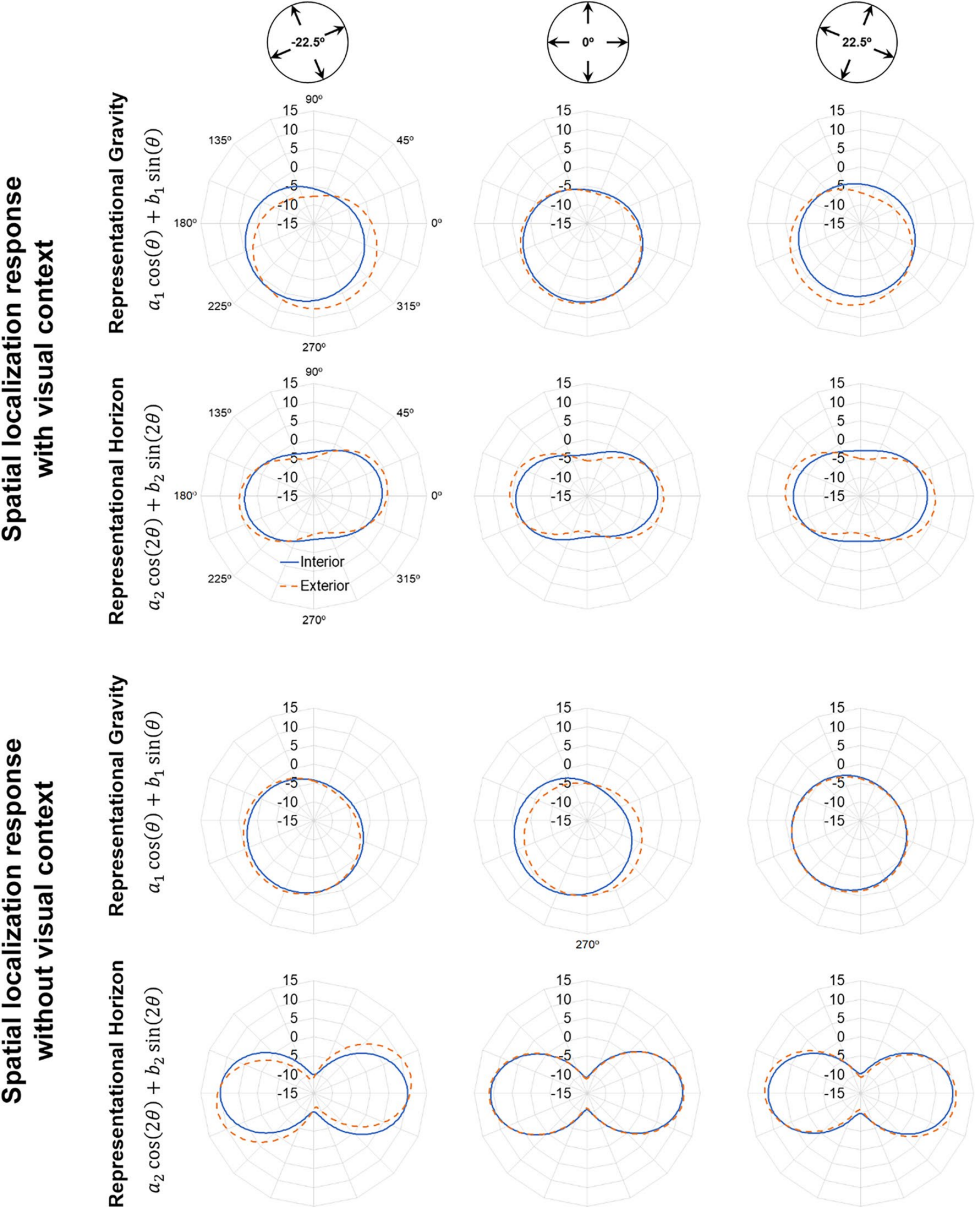
Polar plots of the mean estimated first (coefficients *a*_1_ and *b*_1_—Representational Gravity; RG) and second (coefficients *a*_2_ and *b*_2_—Representational Horizon; RH) harmonic components

Concurrently, a main effect of orientation of the visual context was found for coefficient *b*_*2*_, *F*(2, 76) = 3.24, *p* = .045, partial η^2^ = 0.079, with only a significant linear contrast, *F*(1, 38) = 8.571, *p* = .006, partial η^2^ = 0.184. The latter is of particular relevance, as it results in a trend where, overall, the second harmonic component, indexing Representational Horizon, tends to follow the horizon implied by the visual context (see Fig. 6, bottom plots). Interestingly, coefficient *b*_*2*_ was also modulated by a significant interaction between visual context orientation and scene type, *F*(2, 76) = 5.395, *p* = .006, partial η^2^ = 0.124, with only a significant linear-linear contrast, *F*(1, 38) = 9.048, *p* = .005, partial η^2^ = 0.192, revealing that Representational Horizon’s conformance to the horizon implied by the visual context was chiefly present for exterior, but not the interior scenes (see Fig. 6, dashed lines in the bottom plots for each panel).

The orientation of the visual context was also found to interact with the type of scene in determining the magnitude of the constant *c*, *F*(1.428, 54.273) = 3.896, *p* = 0.039, *partial η*^*2*^ = 0.093. This effect reflects a slight tendency for Representational Momentum to be smaller specifically for upright interior visual contexts. The orientation of the visual context also interacted with response context in modulating the coefficient *a*_*4*_, *F*(2, 76) = 3.762, *p* = .028, partial η^2^ = 0.09, in a pattern in which spatial localisation responses made with an upright visual context resulted in a slightly increased forward displacement for targets moving diagonally. Finally, three-way interactions between response context, visual context orientation, and scene type were found for coefficient *a*_*1*_, *F*(2, 76) = 4.929, *p* = .01, partial η^2^ = 0.115, and *b*_*3*_, *F*(2, 76) = 3.535, *p* = .034, partial η^2^ = 0.085. The former reflects a slight tendency for the direction of Representational Gravity to be biased clockwise (downward and leftward), and more so when the visual context was tilted rightward, albeit solely for exterior scenes and when the spatial localisation response was made with the same visual context present during target motion (see Fig. 5). The latter captures slightly increased forward displacements for targets moving at a downward slant for exterior visual contexts and for interior upright scenes. No other main effects or interactions reached statistical significance.

**Fig. 7:**
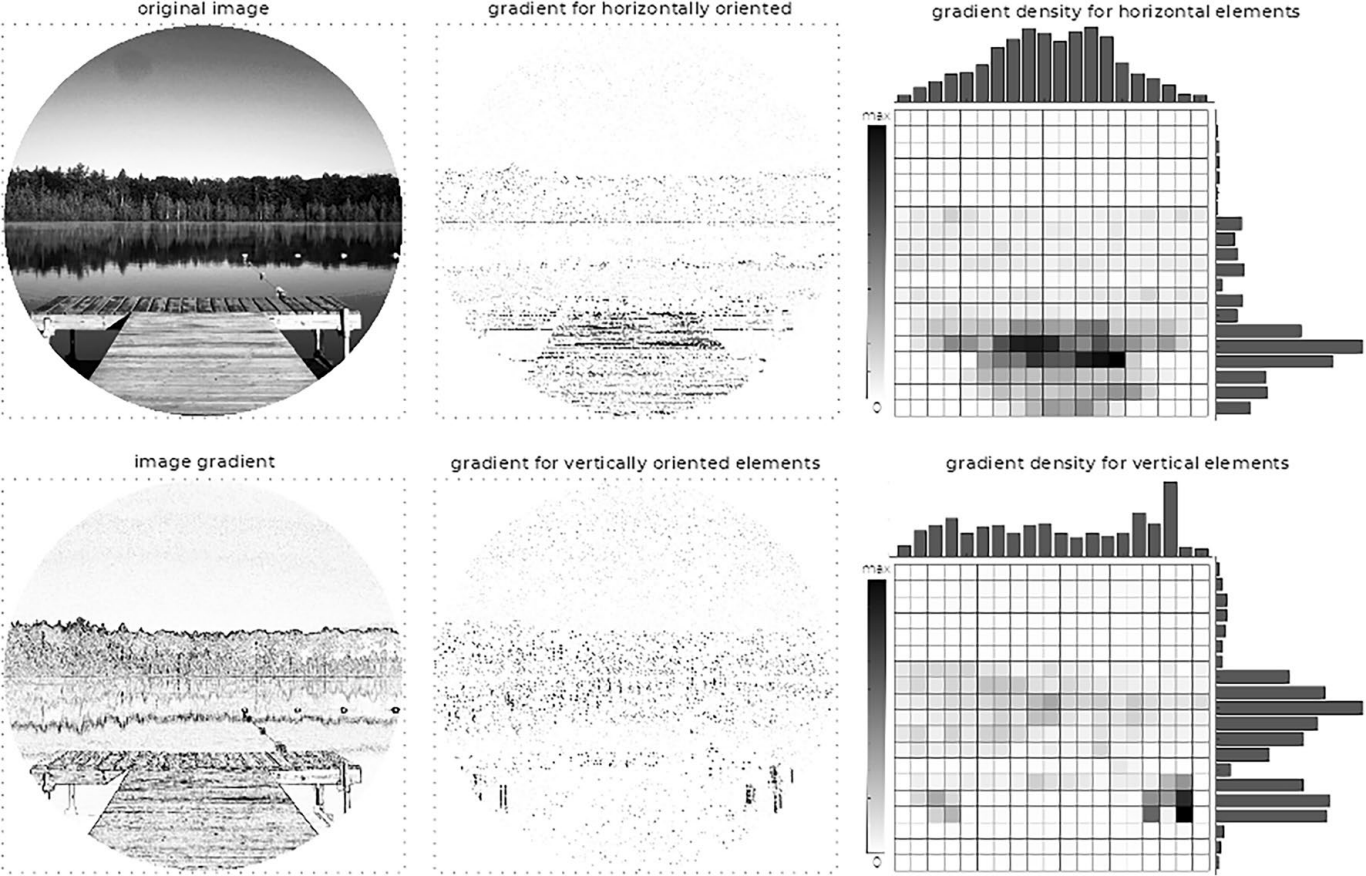
Illustration of the main stages for the overall estimation of horizontally and vertically oriented gradient image elements in the visual context images: the image gradient is computed (leftmost plates), filtered by orientation (centre plates), and a gradient density map computed (rightmost plates). *Note.* To facilitate the illustration and improve readability, the gradient images have been enhanced (luminance inversion and contrast adjustment (i.e., darker values correspond to higher gradients) and the density values normalized for each orientation to cover the full range of the colormap

In light of the disclosed differential effect of scene type on the Representational Horizon, an unplanned *ad hoc* analysis of the image pools was performed, aiming to provide some hints as to which visual features of the exterior scenes, in contrast with interior ones, drive the modulation of Representational Horizon. Arguably, exterior scenes, in general, are more likely to naturally include visible portions of the sky and textured gradients from ground surfaces which, together, strongly and less ambiguously imply a horizon, even if not manifestly shown. Stated differently, we considered the possibility that, by their nature, interior and exterior scenes might present specific visual features regarding horizontally and vertically oriented elements which, in its turn, might play a role in emphasising visual space orientation. To explore this hypothesis, all visual contexts were processed to roughly quantify the presence of such elements (see Fig. 7 for an illustration of the different stages adopted) and to obtain an estimate of the orientation of image elements based upon image gradients.

**Fig. 8:**
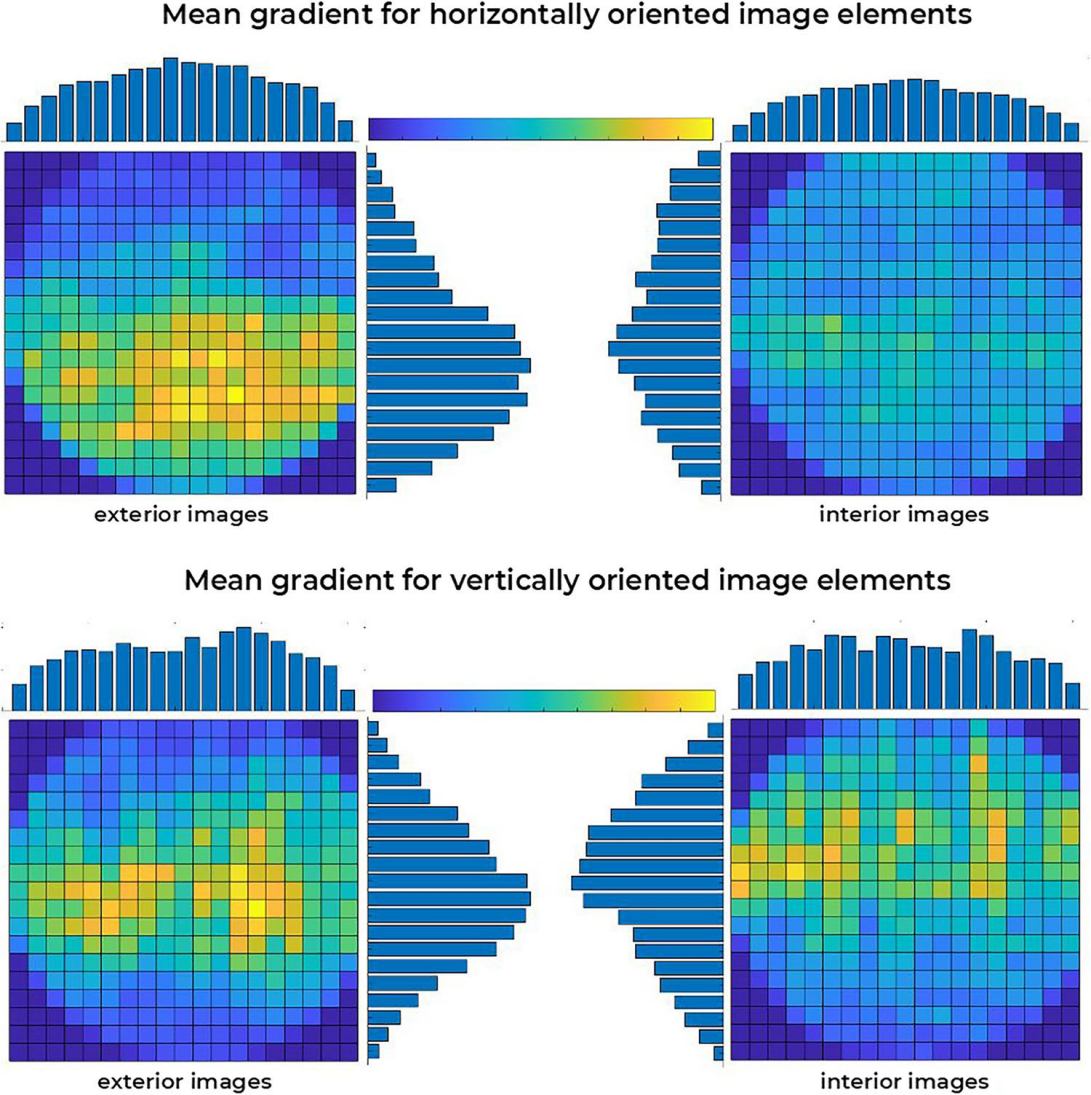
Mean gradient distribution for exterior and interior stimuli regarding horizontally (top row) and vertically (bottom row) oriented image elements. (Colour figure online)

To this end, we computed the image gradients (Burger & Burge, 2016) which yield, at each pixel, the direction of maximum intensity variation along with the rate of that change (a higher gradient magnitude means a more abrupt change). The gradient’s direction was considered to filter only those pixels corresponding to horizontal or vertical variations (corresponding to vertically and horizontally oriented image elements, respectively, e.g., an abrupt luminance change from left to right visually results in a vertical ‘edge’). Finally, the filtered gradient data were processed to compute the amount of gradient per visual context region adopting a 20 × 20 pixels grid. As an overall illustration of the obtained results for each context category and image element orientation, Fig. 8 depicts the mean gradient distribution for horizontally and vertically oriented elements considering all exterior and interior scenes.

To analytically detect differences in mean horizontally and vertically oriented image gradients between exterior and horizontal scenes a mixed MANOVA, given by 20 (scene region columns; repeated measures) × 20 (scene region rows; repeated measures) × 2 (scene type: exterior or interior; between groups).

Considering the context images from left to right, both horizontally, *F*(4.179, 325.99) = 32.143, *p* < .001, partial η^2^ = 0.292, and vertically, *F*(7.807, 608.934) = 7.484, *p* < .001, partial η^2^ = 0.088, oriented image elements were found to vary across scene region columns, in a pattern where both had a more prominent presence near the vertical centre of the visual context. Importantly, neither horizontally, *F*(4.179, 325.99) = 1.326, *p* = .259, partial η^2^ = 0.017, nor vertically, *F*(7.807, 608.934) < 1, oriented image elements were found to be modulated by the type of scene when considering their variation from left to right (compare vertical histograms, above each plot, for both horizontally and vertically oriented image elements, in Fig. 8).

Similarly, mean gradients corresponding to vertically oriented image elements were also found to be modulated by scene region rows (i.e., observing the context images from top to bottom), *F*(3.265, 254.707) = 30.513, *p* < .001, partial η^2^ = 0.281, without significant interaction with scene type, *F*(3.265, 254.707) = 2.445, *p* = .059, partial η^2^ = 0.03. Overall, for both interior and exterior scene pools, vertically oriented image elements were found to be mostly concentrated near the horizontal central regions. Conversely, and importantly enough, observing the mean gradient distribution from top to bottom (i.e., across region rows) unveils a main effect on horizontally oriented image elements, *F*(5.052, 394.029) = 17.877, *p* < .001, partial η^2^ = 0.186, and a statistically significant interaction with the type of scene, *F*(5.052, 394.029) = 4.178, *p* = .001, partial η^2^ = 0.054. The latter effect reflects the fact that horizontally oriented image elements are concentrated on the bottom half region for exterior scenes but more evenly distributed for interior scenes (see horizontal histograms on the interior sides of the top plots in Fig. 8), likely reflecting a higher predominance of a textured visual ground providing horizontal cues which, when contrasting with a less structured sky, in exterior images might strengthen the implication of a well-defined visual horizon.

## Discussion and conclusions

The present experiment aimed to replicate the finding that Representational Horizon, where the spatial localisation of the offset position of a moving target is further displaced beyond what would be expected due to Representational Momentum alone, is biased towards the horizon implied by the visual context (Freitas & De Sá Teixeira, 2021).

In particular, we intended to check for this trend across a wide set of visual contexts, including interior and exterior scenes, as in that previous work only one single visual context was used. The outcomes closely followed our predictions, albeit solely for exterior scenes. This finding was unexpected, for in previous work Freitas and De Sá Teixeira (2021) first reported a tilting of the orientation of Representational Horizon towards the horizon of a visual context using a depiction of an interior scene. However, it should be noticed that the image used as a visual context in that report depicted an empty rectangular frame, with conspicuous horizontal and vertical lines which provided unambiguous visual orientation cues. It can hardly be argued that the set of images employed in the present work, both of interior and exterior spaces, were not rich in visual orientation cues, and it is unlikely that higher-order features, such as the fact that the visual context depicts an open or enclosed space, were responsible for the found difference. However, and by virtue of their very nature, typical exterior scenes might more unambiguously convey a spatial orientation due to the fact that, in such settings, a visually textured ground gradient and a sparsely structured sky are invariably present, besides any other possible elements (e.g., buildings, cars, trees, people), implying a visual horizon line.

Following this reasoning, we set forth to ascertain to what degree the pools of visual contexts employed in our study reflect these natural scene statistics. By quantifying the amount of horizontally and vertically oriented image elements, resorting to image gradients, we found evidence that the former is unequally distributed for exterior but not interior scenes. Specifically, the set of exterior contexts seems to more consistently include a vertical anisotropy, wherein horizontally oriented image elements more likely occur in the bottom half of the scenes, naturally populated by visual surfaces and textured ground. Arguably, this statistical distribution of horizontally oriented image elements results in exterior scenes strongly implying the presence of a horizon line, which, in its turn, might be highly effective in providing a strong spatial orientation cue. Interestingly, this conclusion converges with the finding reported by Hemmerich et al. (2020), where only a simple earth-fixed horizon line was efficient in reducing visually induced motion sickness, supporting the paramount role of that visual feature as a strong cue for spatial orientation. This account opens interesting prospects for future research, where the present experiment might be replicated using as visual context only simple visual features such as a line implying a horizon and/or texture ground gradients (e.g., a chequerboard depicted in perspective).

In any case, the effect of visual context on the orientation of Representational Horizon, in the present study, was shown not to depend on the presence or absence of visual context during the localisation response, revealing that it reflects processes of visual motion representation. However, and unsurprisingly, removing the visual context before the spatial localisation response led to an increase in Representational Momentum, reflecting the fact that participants rely, to some extent, on visual landmarks when providing their spatial localisation judgements (see Gray & Thornton, 2001, for a similar result).

Besides their effect on Representational Horizon, exterior scenes also resulted in a quantifiable increase in Representational Momentum, in comparison with interior scenes. This outcome, in itself, is of particular interest, as it reveals a previously undisclosed trend. To the degree that Representational Momentum reflects the functioning of extrapolating mechanisms for dynamic events (Hubbard, 2005, 2010, 2015, 2019), this effect might reflect tacit knowledge that an enclosed space constrains the trajectory lengths of moving objects—that is, in contrast with an outside setting, where a projectile can move longer and for wider trajectories (given that enough force is imparted to it), an interior space is more likely to be cluttered and any motion is necessarily restricted to the space between the walls (for an effect of explicit barriers, with which the target could collide and bounce back reversing its direction, on the magnitude of Representational Momentum, see Experiment 4 in Hubbard & Bharucha, 1988). Even though somewhat speculative, this account adds to the discussion concerning the degree to which Representational Momentum is sensitive to high-level cognitive expectations (Hubbard, 2006) besides being affected by oculomotor biomechanical constraints (De Sá Teixeira, 2016b; Kerzel, 2000, 2006).

In the present experiment, no particular instruction was given regarding oculomotor behaviour and, thus, it is likely that participants tracked the moving target with their eyes (Churchland & Lisberger, 2002), irrespective of the type of scene used as visual context. Albeit it has been reported that the mere presence of a structured background reduces smooth pursuit eye movements (Collewijn & Tamminga, 1984), it is reasonable to expect no main differences in this regard between our sets of interior and exterior scenes. On the other hand, smooth pursuit eye movements have been shown to be cognitively penetrable, reflecting anticipation of expected trajectories, based on previous experience (Barnes, 2008; Barnes & Collins, 2008; Kowler et al., 2019). Notwithstanding, and since in this study eye movements were not monitored, our explanation remains tentative at this time.

In conclusion, the present study successfully extended the previous finding that the orientation of Representational Horizon, indexed by the second harmonic component underlying the patterns of spatial localisation of a moving target, is biased by visual orientation cues (Freitas & De Sá Teixeira, 2021). Specifically, this biasing was found to be reliably induced by a variety of natural scenes, further emphasising the link between dynamic representations of motion (Freyd, 1987; Hubbard, 2005, 2010, 2015), internal models (De Sá Teixeira & Hecht, 2014; De Sá Teixeira et al., 2013, 2019a, b; Lacquaniti et al., 2013; McIntyre et al., 2001), and spatial orientation (Haji-Khamneh & Harris, 2010; Harris et al., 2011; Howard & Templeton, 1973; Jenkin et al., 2010, 2011; MacNeilage et al., 2008; Mittelstaedt, 1986).
